# Over-speeding trend across self-reported driving aberrant behaviors: A simulator study

**DOI:** 10.3389/fpsyg.2022.1028791

**Published:** 2022-10-06

**Authors:** Alice Grasso, Mariaelena Tagliabue

**Affiliations:** ^1^Department of General Psychology, University of Padua, Padua, Italy; ^2^Department of Civil, Environmental and Architectural Engineering, University of Padua, Padua, Italy; ^3^Mobility and Behavior Research Center—MoBe, University of Padua, Padua, Italy

**Keywords:** driving aberrant behaviors, over-speeding, DBQ, driving simulator, driving style

## Abstract

The aim of the present study is to investigate the relation between self-reported aberrant behaviors as measured by using the Italian version of the Manchester Driver Behavior Questionnaire (DBQ) and actual driving performance during a virtual simulation, focusing particularly on over-speeding. Individual variables are considered based on participants’ behavior, and driving styles are derived from both the self-report questionnaire and the kinematic variables obtained through a moped simulator after the simulated driving task. The experiment was carried out on an Italian sample of 79 individuals aged between 18 and 35 who had to drive throughout virtual road environments. A cluster analysis of the kinematic variables provided by the simulator was used to individuate two different groups of drivers: 45 fell into the cluster named “Prudent” and 34 participants fell into the “Imprudent” cluster. The Prudent participants were characterized by lower acceleration, lower speed, better overall evaluations, and a smaller number of accidents. Correlations showed that self-report responses correlated positively with performance variables in terms of acceleration, speed, and over-speeding. Furthermore, the results from a MANOVA supported and complemented this evidence by emphasizing the usefulness of the integrated approach employed. Overall, these results reflect the suitability of experimental sample-splitting into two clusters, pointing out the appropriateness and relevance of self-report DBQ use with particular emphasis on Ordinary Violations and Lapses. The integrated use of the driving simulator and the self-report DBQ instrument with reference to driving behavior made it possible to support previous theoretical considerations regarding the relations between on-road aberrant behaviors and over-speeding behaviors. It also enabled the addition of evidence on the effectiveness of the simulator in detecting drivers’ actual performance. These results are relevant to allow the integration of useful information to expand intervention and training designs that can be used to reduce risky behavior and promote road safety.

## Introduction

Road mobility trends and driving behavior are relevant topics for ensuring public health. A preliminary classification of the causes of road accidents identified three main classes of causal factors, i.e., individual risk factors, factors related to the safety of road environments and vehicles, and social factors (Rolison, 2020). In the field of road safety, it is relevant to consider driver’s behavior profiles to identify risky attitudes that need to be modified to improve road traffic safety (Ellison et al., 2015). Objective indices focus on studying individuals’ driving performance while using virtual reality simulators or on monitoring naturalistic driving behavior. Subjective indices, on the other hand, refer to individuals’ personal perception of their driving behavior and can be obtained through self-report questionnaires.

An integrated view of driver behavior through the consideration of objective and subjective data may be of particular interest for a comprehensive and detailed understanding of the phenomenon of aberrant driving behavior.

### The role of human factors and misbehaviors in road accidents

The human factor in driving consists of two general components: driving skills or performance and driving style or behavior (Sagberg et al., 2015). Driving skills refer to performance that can improve over time with practice and consolidation of the acquired skill. However, adequate driver skills are not sufficient for safe driving (Taubman-Ben-Ari and Yehiel, 2012). Driving style highlights habitual driving patterns, including speed, headway, and habitual levels of attentiveness and assertiveness, which, according to the ACI report, represent 39.7% of cases of incorrect on-road behaviors in Italy. Among others, inadequate safety distance, drivers’ yielding behaviors approaching pedestrians, and incorrect behaviors of the pedestrian represent, respectively, 7.1%, 3.0%, and 2.7% of the total causes of accidents in 2021 (ACI-Istat, 2022).

In particular, speeding remains a significant problem in many European countries, according to research published by the European Transport Safety Council (ETSC). In fact, the ETSC’s 2019 report shows that a high percentage of vehicles are generally over the speed limits on all types of road—urban roads (by between 35% and 75%, depending on the country), rural roads (by 9–63%), and motorways (23–59%) (ETSC, 2019). In Italy, the full report on road accident data, released by the Istituto Nazionale di Statistica (Istat) with the active cooperation of the Automobile Club d’Italia (ACI) with reference to 2020, shows that, among the most common incorrect driving behaviors (i.e., distraction, failure to yield the right of way, and over-speeding), speeding remains the most frequent, increasing from 9.3% in 2019 to 10% in 2020 (ACI-Istat, 2020), reaching around 13.8% on urban road in 2021 (ACI-Istat, 2022).

The incorrect behaviors just mentioned can fall into two different categories: behaviors implying violation of the road law, or mistakes arising by failure in behavioral planning or monitoring. These two kinks of misbehaviors have different psychological origins, which lead to different ways of intervening in them. Specific training interventions can reduce mistakes, while focusing on changing drivers’ beliefs, norms, and attitudes with regard to driving can reduce violations. A better understanding of accidents and risky behavior on the road can be based on consideration of these classes of driving aberrant behaviors.

Taken together, all these considerations suggest it is appropriate to analyze over-speeding in relation to aberrant driving behavior to reflect on and discuss how exceeding the speed limit while driving can lead to a reduction in headway, reduces the time available to react promptly in situations of potential risk, and increases the probability of driving violations and mistakes. Accordingly, it is interesting to focus studies on the relation between individual variables with reference to over-speeding and aberrant driving behaviors to obtain a detailed understanding of driving behavior.

### Simulated driving

In the present study, we used a driving simulator to identify driving styles through behavioral variables. The issue of the validity of such tools is controversial, due to the varieties of simulators and scenarios, methodologies, tasks, and variables employed in dedicated studies, beside the range of considered outcomes which make the results hardly comparable (Helland et al., 2013). Studies supporting the usefulness of driving simulators for research in the field of traffic psychology emphasize advantages such as the possibility to observe driving behaviors in risky conditions, that can lead to accidents, without incurring in real dangers, the fact that simulators allow reproducibility and standardization of conditions, and the possibility to control traffic environment (de Winter et al., 2012).

On the other hand, disadvantages related to motion and physical or visual fidelity suggest caution in data interpretation (Wynne et al., 2019), limiting the generalizability of results to the on-road environment. Moreover, Martín-delos Reyes et al. (2019), in their systematic review, found no evidence in favor or against the efficacy of driving simulator, pointed out that studies focused on testing the effect of trainings are heterogeneous and suffer of several drawbacks, mainly related to sample size, recruitment based on voluntary participants, dropouts that may lead to selection bias, and the use of non-blinded self-reported measures. Other limits are sampling biases, dropouts caused by simulator sickness, and predictive validity (de Winter et al., 2012).

However, similarities between simulator performance and on-road behaviors for some variables, such as attention to traffic lights and stop signals, space exploration, speed maintenance (especially at intersections), and mirror monitoring have been shown by Meuleners and Fraser (2015), and similarities between simulated and on-road driving have been demonstrated in testing fitness to drive under the effect of drug or alcohol (Veldstra et al., 2015; Jongen et al., 2016).

Taking into account the pros and cons just mentioned, we reasoned that the observation of correlations between simulated driving performance and self-reported measures may be useful in contributing to provide converging evidence (to join with those from on-road observations) of the usefulness of the DBQ for driving style assessment.

### Development and validation of the driver behavior questionnaire

Individual’s attitudes and personal variables constitute essential elements in determining the individual’s specific behaviors and actions in the surrounding environment. An individual’s intentional conduct is based on constant monitoring and evaluation of risks and benefits of a possible action. Consequently, it is important to focus on personality, cognitive, temporal, and motivational variables which may lead to a lack of accuracy in analyzing actions and their consequences at the individual and community levels. Adequate risk perception directly influences attitudes toward road safety, leading to a greater adherence to traffic rules and an increased sense of responsibility while driving as well as discouraging secondary aggressive or careless behavior on the road (Ram and Chand, 2016).

Reason et al. (1990) focused on the analysis of unsafe driving behavior in relation to two classes of aberrant behaviors: unintentional mistakes and intentional violations. Mistakes relate to driving skills, as they involve cognitive failures in information processing; violations are related to driving styles because of their psychosocial and motivational aspect and consequently can only be understood within a specific social context. In this regard, Reason et al. (1990) developed the Manchester Driver Behavior Questionnaire (DBQ) in the UK for the study of aberrant self-reported drivers’ behavior. The original version of the questionnaire contained 50 items identifying three classes of behavior: violations, errors, and lapses. The original publication was based on a sample of 500 drivers aged between 20 and 56 who were asked to rate the frequency with which they performed risky driving behaviors on a Likert scale ranging from 0 (never) to 5 (nearly all the time). Over the past decades, numerous researchers have developed, modified, and updated new versions of this measurement instrument in various countries, showing significant differences in factor structure, number of items, and reference populations. A shortened 24-item version of the instrument was developed, including, for each class of behaviors, the eight items with the highest loadings as shown in the original study (Parker et al., 1995). This version of the questionnaire was characterized by a three-factor structure which confirmed the distinction between errors, lapses, and violations and showed a satisfactory test–retest reliability (*r* = 0.75 for the lapse scale; *r* = 0.81 for the violation scale; *r* = 0.69 for the error scale; overall coefficient = 0.78). Moreover, Parker et al. (1995) demonstrated that DBQ violation scores are good predictors of accident rates. Further studies, focusing on violations and errors, contributed to the distinction between ordinary and aggressive violations defining the former as the violations acted for instrumental purposes and the latter as hostile behaviors toward other drivers (Lawton et al., 1997). The distinction between the two types of violation was also confirmed by Parker et al. (1998). The shortened versions of the questionnaire by Parker et al. (1995), updated with the extension of the violation scale provided by Lawton et al. (1997), led Lajunen et al. (2004) to develop a 27-items version of the questionnaire, validated in a cross-cultural study which compared Dutch, British and Finnish samples. The comparison between the three samples showed alpha reliability coefficients, for the four scales in all the considered samples, comparable with those found in previous studies. Moreover, the factor analysis revealed the presence of four first-order factors corresponding to the four categories of aberrant behaviors discussed (aggressive violations, ordinary violations, errors, and lapses) which, in turn, can be grouped into the two second-order factors, that is, violations and mistakes, thus confirming the theoretical framework of the original proposal (Reason et al., 1990). This factorial structure was also confirmed by Smorti and Guarnieri (2016) who validated the Italian version of the questionnaire, employed in the present study (see the Methods section for the details).

In all the studies mentioned, the authors highlighted the crucial implications of using the DBQ in study on road safety, in that the possibility of the questionnaire to distinguish between aberrant on-road behaviors with different psychological origins would allow to target interventions in a better way. Specifically, being aggressive violations driven by affective attitudes Parker et al. (1998), road safety interventions on group of drivers with high scores in this scale should point to induce changes in beliefs toward aggressive driving by promoting, for instance, the idea of cooperation, rather than competition (Lawton et al., 1997), or through training aimed at improving the ability to manage stress and frustration. Conversely, drivers who tends to commit errors may benefit from attentional trainings.

To summarize, the DBQ constitutes an important measurement tool that contributes to highlight the relation between these classes of aberrant road behavior and driving behavior, with particular reference to risky driving, aggressive behavior on the road, drivers’ risk-seeking, and the occurrence of accidents (Smorti and Guarnieri, 2016).

Investigating the relation between these behaviors and over-speeding on the road can provide a better understanding of individual factors underlying driving behavior, enabling the development of more targeted and effective safety education programs and road-traffic management interventions (Wang and Xu, 2019).

The aim of the present study is to determine whether the results of a driving-style questionnaire are in agreement with driving behavior in terms of over-speeding based on the considerations derived from the literature, considering the factorial structure of the DBQ described above. For these reasons, we decided to analyze the relation between self-reported driving aberrant behaviors and actual over-speeding behaviors as observed during a simulated driving task through a moped-driving simulator. Participants’ individual variables are taken into account on the basis of their behavior, and driving styles derived from the integration of subjective data obtained from the self-report questionnaire and objective performance data. The hypotheses regarding the importance of investigating the presence of correlations between subjective and objective data were focused on assessing the coherence between the results obtained by the DBQ and participants’ actual speeding behaviors during the virtual driving session. Accordingly, it was expected that questionnaire scores identifying risky on-road behaviors would be positively correlated with objective variables related to risky and reckless virtual driving performance, showing a correspondence between subjective and objective data on participants’ driving styles and behaviors. Specifically, considering the role speeding behaviors play in accounting for a great number of accidents, we expected to find evidence of correlations between driving aberrant behavior as attested by the DBQ scores and the kinematic variables derived by the driving performance related to participants’ attitudes toward speed control.

Identifying such possible correspondences may be useful for a better understanding of the phenomenon of road accidents and aberrant driving behavior, which, in turn, can encourage the design of intervention projects tailored to the specific characteristics of individuals’ driving styles.

## Materials and methods

### Instruments

The experiment was carried out at the Department of General Psychology of the University of Padua. Participants had to fill out an online questionnaire (provided through the Google Form module), which required approximately 10 min, and to complete a simulated driving session with the Honda Riding Trainer (HRT) simulator lasting approximately 15 min.

#### The questionnaire

The online questionnaire was structured in two sections. The first battery of questions was intended to obtain the participants’ biographical data, information on driving licenses, and on-road driving experience. The second battery referred to the DBQ instrument in its reduced Italian version of 27 items (Smorti and Guarnieri, 2016), to which participants had to provide answers on a Likert scale from 0 (never) to 5 (nearly all the time), indicating how often they have found themselves in the type of situations presented by the items during the last year. In the validation study of Smorti and Guarnieri, the four latent variables of the model showed high correlation values, suggesting the presence of two second-order factors: Violations, consisting of the factors Aggressive Violations and Ordinary Violations and derived from the mean of the values obtained by the responses to the related sub-scales; unintentional Mistakes, consisting of Errors and Lapses and derived from the mean of the values obtained from the responses to these last two sub-scales. As to the four latent variables, aggressive Violations involve an aggressive interpersonal component that reflects an affective and emotional character and are derived from the mean scores of three items. Ordinary Violations include intentional actions that deviate from safe driving without a specific aggressive purpose and are derived from the mean scores of eight items. Errors represent failures in action planning due to misinterpretation of a problem or its solution and are derived from the mean scores of eight items. Finally, Lapses indicate actions that may unintentionally deviate from the original intention and are derived from the mean scores of eight items (for the list of the items, see Smorti and Guarnieri, 2016). The Italian validation study showed better alpha reliability coefficients than those obtained in the cross-cultural study by Lajunen et al. (2004) both for the first order (Aggressive Violations = 0.72; Ordinary Violations = 0.84; Errors = 0.87; Lapses = 0.83) and the second order (Violations = 0.87; Mistakes = 0.90) factors. As to the concurrent validity, Smorti and Guarnieri (2016) reported significant correlations (ranging from 0.21 to 0.63) between the first and second order factors of the DBQ, three scores of dangerous driving (from the Dula Dangerous Driving Index—Italian version; Chiorri et al., 2008), and one score of thrill and adventure seeking (Italian version of the thrill and adventure seeking subscale of the Sensation Seeking Scale-V, validated by Manna et al., 2013). The authors also concluded that despite the correlations between the DBQ factors and self-reported accidents are low (range 0.13–0.18), albeit significant, they are in line with the findings of previous studies (de Winter and Dodou, 2010; de Winter et al., 2015).

#### The driving simulator

The HRT was built and designed to train people to drive safely by exposing them to potentially dangerous situations based on the most common cases of accidents that can occur in real life (MAIDS report, 2004 on accidents in Europe). It has been widely employed to assess relations between driving styles in novice drivers and their sensation-seeking and decision-making abilities (Gianfranchi et al., 2017b), adolescents’ beliefs about peer behaviors on the road (Gianfranchi et al., 2018), and how psychophysiological reactivity to a risky scene modulates driving behaviors (Tagliabue et al., 2019).

The simulator allows to place participants in simulated risky situations (in which, for instance, a car suddenly opens a door, pedestrians cross without looking, or vehicles overtake on the wrong side of the road, slow down, or turn unexpectedly without a turn signal) and records kinematic driving variables of the participants’ performance at a sampling rate of one frame every 0.03 s. The simulator looks like a moped and consists of a seat, handlebars, and a monitor connected to a PC. Two loudspeakers reproduce typical road noises and give instructions on how to use the tool and the route to follow during the driving simulation. The HRT tool is useful for evaluating participants’ simulated driving performance in various degrees of road risk exposure to identify driving styles with specific behavioral patterns. The simulator offers various types of routes and options with regard to vehicle type, driving mode, and environmental conditions. The roads to be driven can be main, secondary, or tourist roads. The driving mode can be manual with the use of pedals or automatic, and the environment can represent the route in daytime, at night, or with fog (Gianfranchi et al., 2017a; for a list of possible scenarios provided by the simulator, see also the supplementary material in Tagliabue et al., 2017).

### Procedure

The virtual driving session was conducted in the laboratory using the HRT moped-driving simulator. Participants filled out the informed consent form and then took part in the simulation session. During the driving session, each participant read a document with instructions and was informed about the use of voice prompts to indicate what to do during the driving session and how to carry out the session itself. The researcher attending the simulation was in charge of making sure that each participant understood the information provided and was responsible for showing the participants the commands to turn on the simulator and to drive through the simulation. Following these informative instructions, the researcher allowed the participants to practice with a brief scenario lasting approximately 3 min, so as to get acquainted with the instrument controls on a route with free navigation and without the presence of other vehicles. Then, the researcher administered the two experimental routes in daytime mode. During the driving session, two scenarios were administered to participants, each including eight risky scenes; they were instructed to follow the voice prompts that indicated how to continue the specific route on the road, respect the traffic code, and behave as if they were driving on a real road. The research was conducted with approval by the Ethical Committee for the Psychological Research of the University of Padua.

### Participants

The experiment was carried out on a sample of 79 volunteer participants from November 2021 to February 2022. The inclusion criteria were having a car or motor driving license for at least 6 months, an age between 18 and 35 years, and driving experience of at least 1,000 km per year. Participants with previous experience with driving simulators were excluded. The sample of participants, consisting of 32 men and 47 women, had an average age of 21 years (range 18–29). All the participants were unaware of the purpose of the experiment.

### Experimental design and data analyses

Data analysis was conducted using the IBM SPSS Statistics tool version 28.0.1.0 (142). First, the statistical tool was used to identify Pearson correlations between the answers to the DBQ and the data obtained from the use of the HRT simulator. The six variables of the DBQ were taken into account: the mean of ordinary violations (mean OV), the mean of aggressive violations (mean AV), the mean of errors (mean E), the mean of lapses (mean L), the mean of violations (V; mean between ordinary and aggressive violations), and the mean of mistakes (M; mean between errors and lapses). These variables were correlated with the 18 simulator variables extracted from each participant’s log file namely: the mean of acceleration (M_Acceleration_), the standard deviation of acceleration (SD_Acceleration_), the number of times the front brake was used (N_FBrake_), the mean of front brake use (M_FBrake_), the standard deviation of front brake use (SD_FBrake_), the number of times the rear brake was used (N_RBrake_), the mean of rear brake use (M_RBrake_), the standard deviation of rear brake use (SD_RBrake_), the mean speed (M_Speed_), the standard deviation of speed (SD_Speed_), the time the participant spent over the speed limit in terms of number of frames of the HRT log file (NFr_Over-speeding_), the number of instances of over-speeding (N_Over-speeding_), the mean of instances of over-speeding (M_Over-speeding_), the highest value of over-speeding (Max_Over-speeding_), the mean of on-road instability (M_Instability_), the standard deviation of on-road instability (SD_Instability_), the number of accidents (Accidents), and the overall evaluation (Evaluation) of driving performance provided by the simulator (lower numerical values refer to safer performance).

With regard to the driving performance evaluations provided by the simulator, 4-value scores were provided to indicate the degree of risk of the participants’ behaviors for each of the 16 scenes of the session (A = 1, B = 2, C = 3 or D = 4), with the lower numerical value referring to safer performance and the higher value referring to the occurrence of an accident. The total score of these evaluations was calculated by the mean of evaluations of each scene in each participant’s log file to obtain the overall simulation performance profile.

Second, the SPSS statistical tool was used to extract the driving style according to the kinematic variables provided by the simulator throughout a cluster analysis and to conduct a MANOVA on DBQ scores and speeding HRT variables with the variable Cluster as the between-participants factor.

Concerning the cluster analysis, a non-hierarchical *k*-means cluster analysis was performed on the *z*-scores of the driving parameters, using the centroids identified in a previous visual feedback study as a reference. In fact, the present study represents the second part of previous research conducted with the same HRT simulator intended to investigate the effectiveness of an alert system that provided simultaneous visual feedback when the speed limit was exceeded during a driving simulation and the persistence of its effect over a period of one month (Tagliabue et al., 2021). The aim of the cluster analysis was to identify prudent and imprudent drivers, as in the reference study.

Finally, a MANOVA with the variable Cluster as the between-participants factor was conducted on the sample of 79 participants, considering 14 dependent variables, including kinematic variables related to speeding behaviors and scores obtained with the DBQ. The list of variables is as follows: the mean of acceleration (M_Acceleration_), the standard deviation of acceleration (DS_Acceleration_), the mean speed (M_Speed_) the standard deviation of the speed (SD_Speed_), the time the participant spends over the speed limit (NFr_Over-speeding_), the number of instances of over-speeding (N_Over-speeding_), the mean of instances of over-speeding (M_Over-speeding_), the highest value of over-speeding (Max_Over-speeding_), the mean of ordinary violations (mean OV), the mean of aggressive violations (mean AV), the mean of errors (mean E), the mean of lapses (mean L), the mean of violations (V; mean between ordinary and aggressive violations), and the mean of mistakes (M; mean between errors and lapses).

## Results

Table 1 reports the significant Pearson correlations between the DBQ variables and the HRT simulator variables. HRT variables that did not show any significant correlation with the DBQ scores are not included. As can be seen, Aggressive Violations correlated positively with the standard deviation of acceleration and with the mean of over-speeding. Ordinary Violations correlated positively with all the variables related to speed and over-speeding. Globally, overall Violations were found to be positively correlated with the same HRT variables that were positively correlated with the mean of Ordinary Violations, with the exception of the number of instances of over-speeding. Errors seem uncorrelated with the speeding variables considered. Lapses were negatively correlated with the mean of rear brake use and positively correlated with the mean of speed, the time the participant spent over the speed limit, and the mean of over-speeding. Globally, Mistakes were found to be positively correlated with the time the participants spent driving over the speed limit.

**Table 1:** The table shows *r* coefficient values derived from Pearson correlations between the DBQ variables and the HRT simulator variables which showed significant correlations.

	Mean AV	Mean OV	Mean E	Mean L	V	M
DS_Acceleration_	0.253*	0.249*	0.175	0.162	0.296*	0.191
M_RBrake_	−0.029	−0.134	−0.087	−0.273*	−0.092	–0.212
M_Speed_	0.208	0.300**	0.068	0.254*	0.296**	0.191
NFr_Over-speeding_	0.209	0.356**	0.127	0.278*	0.327**	0.236*
N_Over-speeding_	0.090	0.239*	0.112	0.189	0.189	0.174
M_Over-speeding_	0.234*	0.325**	0.068	0.224*	0.326**	0.172
Max_Over-speeding_	0.193	0.223*	0.009	0.167	0.243*	0.106
*N*	79	79	79	79	79	79

*p < 0.05; **p < 0.01.

As reported before, a non-hierarchical *k*-means cluster analysis was carried out on the *z*-scores of the driving parameters of the simulator. In this way, participants were distributed into two clusters. Specifically, 45 participants (of whom 17 were men) fell in the Prudent cluster, whereas 34 participants (of whom 15 were men) fell in the Imprudent cluster of drivers.

**Figure 1:**
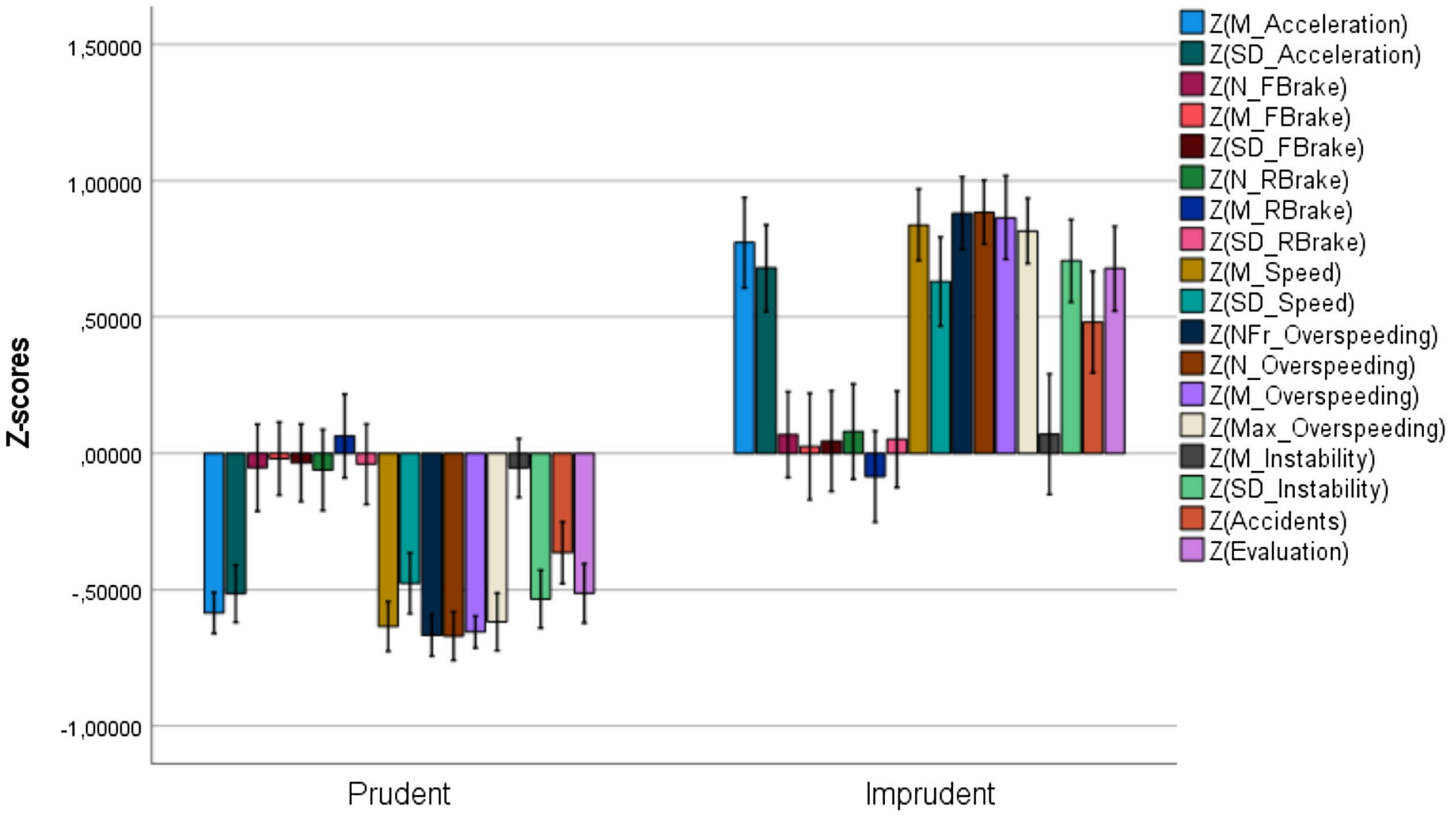
*Z*-scores of the HRT kinematic variables in the two clusters. Vertical bars represent standard errors.

As can be seen in Figure 1, Prudent drivers showed lower M and SD of Acceleration, lower values in all the variables related to speeding behaviors and in the SD of Instability, fewer accidents, and better overall Evaluations (lower values indicate a better performance, as explained above).

Regarding the MANOVA, at the multivariate level, the Cluster factor was statistically significant, with *F*(12,66) = 11.38, *p* < 0.001, Wilks *λ* = 0.33.

At the univariate level, significance is present for the following dependent variables: M_Acceleration_ with *F*(1,77) = 64.82, *p* < 0.001, $\eta_{p}^{2}$ = 0.46; SD_Acceleration_ with *F*(1,77) = 42.06, *p* < 0.001, $\eta_{p}^{2}$ = 0.35; M_Speed_ with *F*(1,77) = 89.59, *p* < 0.001, $\eta_{p}^{2}$ = 0.54; SD_Speed_ with *F*(1,77) = 33.45, *p* < 0.001, $\eta_{p}^{2}$ = 0.30; NFr_Over-speeding_ with *F*(1,77) = 112.79, *p* < 0.001, $\eta_{p}^{2}$ = 0.59; N_Over-speeding_ with *F*(1,77) = 115.24, *p* < 0.001, $\eta_{p}^{2}$ = 0.60; M_Over-speeding_ with *F*(1,77) = 103.16, *p* < 0.001, $\eta_{p}^{2}$ = 0.57; Max_Over-speeding_ with *F*(1,77) = 79.97, *p* < 0.001, $\eta_{p}^{2}$ = 0.51; AV with *F*(1,77) = 4.11, *p* < 0.05, $\eta_{p}^{2}$ = 0.05; OV with *F*(1,77) = 5.85, *p* < 0.05, $\eta_{p}^{2}$ = 0.07; L with *F*(1,77) = 5.72, *p* < 0.05, $\eta_{p}^{2}$ = 0.07; V with *F*(1,77) = 6.93, *p* < 0.05, $\eta_{p}^{2}$ = 0.08; M with *F*(1,77) = 4.23, *p* < 0.05, $\eta_{p}^{2}$ = 0.05.

## Discussion

### Relation between driving performance and DBQ scores

As shown above, the results obtained from the Pearson correlation matrix between DBQ variables and HRT variables provide important insights. First, the overall view suggests that the DBQ’s four latent variables and two second-order factors correlate with seven out of 18 HRT-simulator kinematic variables, all related to speeding behaviors. This first consideration is coherent with our hypothesis of a strict connection between aberrant driving behaviors and attitudes toward speed control. In detail, the positive correlations involve the overall Violation scores more than the overall Mistakes scores. As to the former, participants who made a greater number of Violations seemed to drive more quickly (higher M_Speed_) and show less regular control of acceleration (higher SD_Acceleration_). Thus, they spent more time exceeding the speed limit and showed a higher mean and the highest values of over-speeding. However, more declared Mistakes on the DBQ were only linked with a greater amount of time spent over the speed limit. Again, the results regarding Violations seem mainly due to Ordinary, rather than Aggressive Violations, even though the latter reflects on variability in Acceleration behaviors and mean of over-speeding. The overall Mistake results were exclusively due to Lapses (that is, unintentional deviation from voluntarily planned actions), rather than failure in planning the correct behavior. On the basis of existing literature, these results show how Violations are related to driving styles due to their psychosocial and motivational aspects; consequently, Violations can only be understood within a specific social context, including aspects of Ordinary and Aggressive Violations (Smorti and Guarnieri, 2016). Therefore, the greater tendency to report (through self-report questionnaires) Violations during driving experiences is reflected in the greater propensity to engage in risky and impulsive behavior in simulated driving. In fact, the simulator variables involved in these correlations show attitudes and performances that are potentially hazardous to road safety and that can be linked to less conscientiousness, greater extraversion in terms of sensation-seeking and risk-taking, and greater nervousness with reference to the tendency to make greater variations in acceleration while driving, which can also show an impetuous attitude characterized by unpredictability. The DBQ’s second-level factor of overall Violations is the result of the mean score of questionnaire items closely related to acceleration, speed, and over-speeding, with particular emphasis on items 9, 10, 20, and 27. Item 9 refers to “Uscire da un incrocio così velocemente da obbligare un altro conducente che avrebbe la precedenza a fermarsi per farti passare” [Pull out of a junction so far that the driver with right of way has to stop and let you out] (Smorti and Guarnieri, 2016, p. 17); item 10 asks the participant to rate the frequency of the behavior “Non rispettare i limiti di velocità su una strada residenziale” [Disregard the speed limit on a residential road] (Smorti and Guarnieri, 2016, p. 17); item 20 focuses on “Partire a tutta velocità davanti a un semaforo con l’intenzione di ‘battere’ il conducente accanto” [Race away from traffic lights with the intention of beating the driver next to you] (Smorti and Guarnieri, 2016, p. 18); and item 27 refers to “Non rispettare i limiti di velocità in autostrada” [Disregard the speed limit on a motorway] (Smorti and Guarnieri, 2016, p. 18). Consequently, the resulting correlations support the validity of this DBQ factor, corresponding with the actual individual’s driving performance.

To sum up, the correlations between overall Violation scores and over-speeding parameters highlight extremely risky and dangerous driving behavior due to the overall speeding, and are coherent with the literature showing that the propensity to commit violations is linked to accident involvement (Parker et al., 1995). In addition, the correlation between the mean of Violations and the time the participants spent over the speed limit can be interpreted by the fact that the longer the driver maintained a speed over the road limit, the greater the likelihood of incurring traffic violations. High scores in the mean of Ordinary Violations correspond to high scores of mean of Speed, and the time the participants spent over the speed limit. Furthermore, the mean of Ordinary Violations correlates positively with the standard deviation of Acceleration, the number of instances of over-speeding, and the highest value of over-speeding. These positive correlations show the specificity of the DBQ variables with regard to Ordinary Violations in correlating with aspects of driving performance in terms of acceleration, speed, highest value of over-speeding, and driving time over the speed limit, which is reflected in the above considerations with regard to the mean of overall Violations (second-order DBQ factor). Parallel to these considerations, results show that high scores in the mean of Aggressive Violations correspond only to higher variability in Acceleration and higher mean values of over-speeding. These results show that the over-speeding phenomenon is not necessarily due to aggressiveness. However, it can be reflected in two behavioral variables, suggesting that a greater variation in acceleration and a higher mean of over-speeding can be interpreted as greater impulsiveness and nervousness in terms of aggressive driving behavior related to affective and emotional attitudes toward a certain amount of violence, which can lead to greater impairment in road safety. In other words, even if the relation between speeding and accidents is not direct, it is plausible that the more the over-speeding, the time spent in speeding, the average and the extent of the over-speeding, the greater the probability to incur in a crash.

Driving Mistakes’ positive correlation with the time participants spent over the speed limit agrees with what has been stated in the literature in terms of DBQ’s second-order factor structure, considering that Mistakes relate to driving skills as they involve cognitive failures in information processing. In this way, the correlation between the mean of Mistakes and the time the participants spent over the speed limit can be interpreted by focusing on the fact that drivers’ mistake in keeping their speed under control can be linked to distraction and failures in assessing their own performance while driving, thus contributing to an increase in the likelihood of aberrant driving behaviors. Analogously, Lapses corresponding to high levels of the mean of speed, the time the participants spent over the speed limit, and the mean of over-speeding seem to indicate that the greater propensity for driving actions to unintentionally deviate from the driver’s original intention is linked to greater driver distraction. It is worth noting that in the study of Parker et al. (1995) lapses did not correlate with accidents. This may suggest that speeding propensity due to difficulty to control the execution of the correctly planned actions is less dangerous than speeding misbehaviors of drivers who tend to commit more ordinary violations, being the latter deliberate behaviors caused by the choice not to respect traffic rules or bad attitudes toward road laws (Reason et al., 1990). Instead, the lack of correlations between DBQ Error scores and speeding variables is in line with the results by Parker et al. (1995) who demonstrated that violations, but not errors, predict self-reported accident involvement, considering the causal role of speeding in crashes showed by international statistics.

Finally, note that all the significant correlations reported are positive, with the exception of the negative correlation between the scores of Lapses and the mean of rear brake use obtained from the HRT instrument. This negative correlation indicates that high scores referring to a participant’s tendency to make driving lapses are related to a lower tendency of the same participant to use rear brakes during the driving simulation. It must be said that rear brake performance depended on the use of the left brake lever of the simulator’s handlebar, while the front brake lever is positioned on the right, near the throttle grip. Consequently, participants may have been induced to keep the two components of the so-called “celeration” behaviors (acceleration and deceleration) separate—that is, the two tasks of acceleration and braking—by dedicating one hand to one task and the other hand to the other task, thus using the two levers alternately. In this case, the value reported by the simulator with reference to the rear brake can be considered an expression of overall brake use. If so, it is possible to reflect on how individuals who are more inclined to make Lapses while driving may be less conscientious in terms of distraction so as to omit actions that are important for safe driving with reference to brake use. Brake use refers to a driver’s careful analysis and evaluation of risks and benefits on the road in terms of road speed and compliance with the rules for the safety of oneself and others. Thus, reduced brake use may refer to less agreeableness, or less willingness to cooperate and respect social norms for community well-being and less sense of rigorous planning of one’s own road behavior. However, the fact that it does not correlate with Violations, but only with Lapses, seems to indicate that (at least in our sample) its under-use seems mainly due to a difficulty in carrying out correctly planned actions.

### Objective and subjective data trend across the two clusters of the experimental sample

The results obtained from the MANOVA proved to be important for further discussions on data analysis. On a multivariate level, the results show significant differences between the two clusters, Prudent and Imprudent, into which the sample of 79 participants was divided. These differences applied not only to the HRT variables of acceleration, speed, and over-speeding recorded during the driving session (that can be a consequence of the method used to identify the two different driving styles), but also with reference to the DBQ scores. This result supports the appropriateness of sample division into the two clusters, showing that the differences found in the objective data obtained through the HRT simulator according to the cluster to which participants belong overlap with the differences highlighted through the DBQ’s subjective information, which shows habitual driving behavior due to individual variability of each participant. In line with these considerations, the use of the two instruments seems appropriate with regard to the individual use of each instrument and for the integrated use of the two tools to derive detailed information on individuals’ driving behavior.

**Figure 2:**
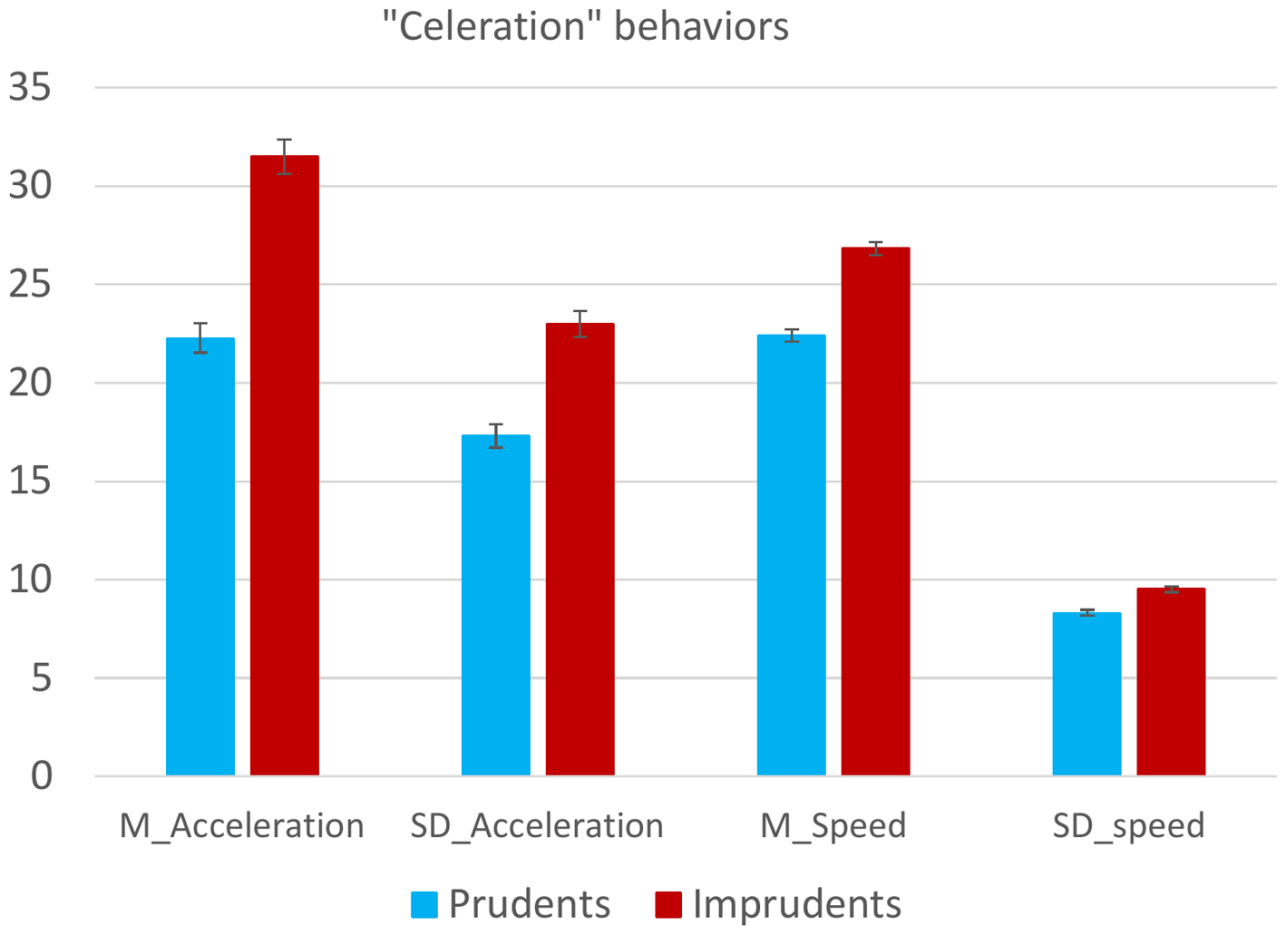
The figure shows the trends in differences in celeration behaviors between Prudent and Imprudent participants. Vertical bars represent standard errors.

In detail, the results at the univariate level show that the type of cluster to which participants belonged significantly influenced the differences in their performance in terms of the mean values provided by the simulator, including all the variables related to acceleration, speed, and over-speeding. Specifically, as seen in Figure 2, Imprudent participants showed higher values in all the celeration variables.

**Figure 3:**
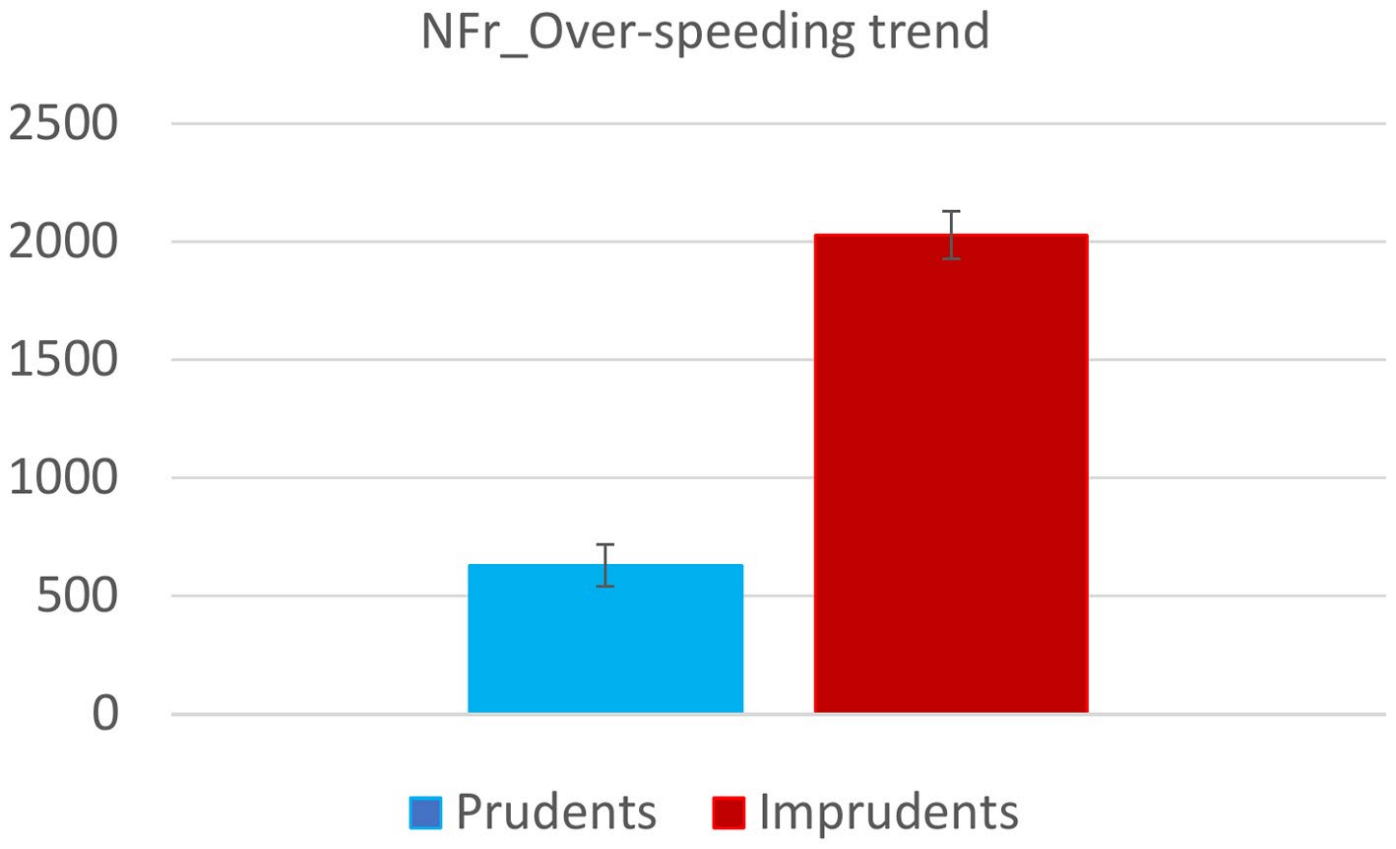
The figure shows time (in terms of number of sample frames) spent exceeding the speed limit by Prudent and Imprudent participants. Vertical bars represent standard errors.

**Figure 4:**
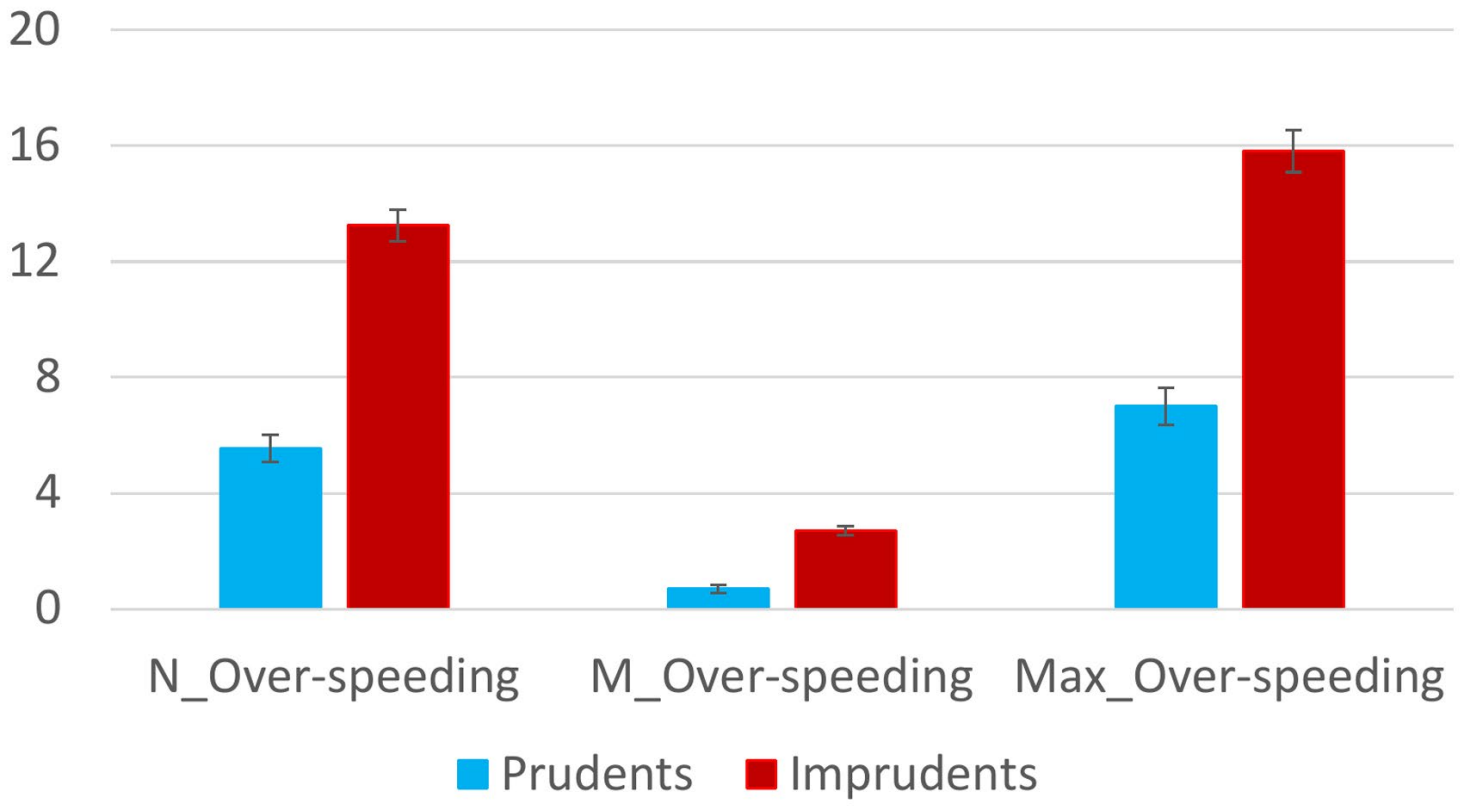
The figure shows over-speeding trends in Prudent and Imprudent participants. Vertical bars represent standard errors.

Moreover, the same trend is observed for time spent driving faster than the speed limit (see Figure 3) and number of instances, mean, and maximum value of over-speeding (see Figure 4).

That seems in line with the idea that celeration behaviors, which are crucial for road safety, play a role in defining one’s driving style, further supporting the appropriateness of this sample subdivision into the two clusters on the basis of driving behavior, albeit simulated.

The variables considered are of particular interest, considering the importance of emphasizing how over-speeding is linked to risky driving performance. These considerations can be supported by literature data, such as those collected in studies on associations between driving styles and Big Five personality factors, to derive a more comprehensive understanding of road behavior (Taubman-Ben-Ari and Yehiel, 2012). Risky, angry, dissociative, and high-speed driving styles are associated with low scores on the personality factors of conscientiousness and agreeableness. These styles turned out to be linked to high extraversion scores due to strong sensation-seeking tendencies and worse risk perception while driving. In fact, these styles have been linked to a greater tendency to be daring and assertive, with less tolerance and concern for others (Taubman-Ben-Ari and Yehiel, 2012). When driving, this attitude can lead to increased acceleration, speeding, and over-speeding, increasing the likelihood of driving risks as an expression of imprudence found in the Imprudent participants during the driving session. Consequently, simulator variables related to over-speeding seem relevant and appropriate to detect such individual driving attitudes.

**Figure 5:**
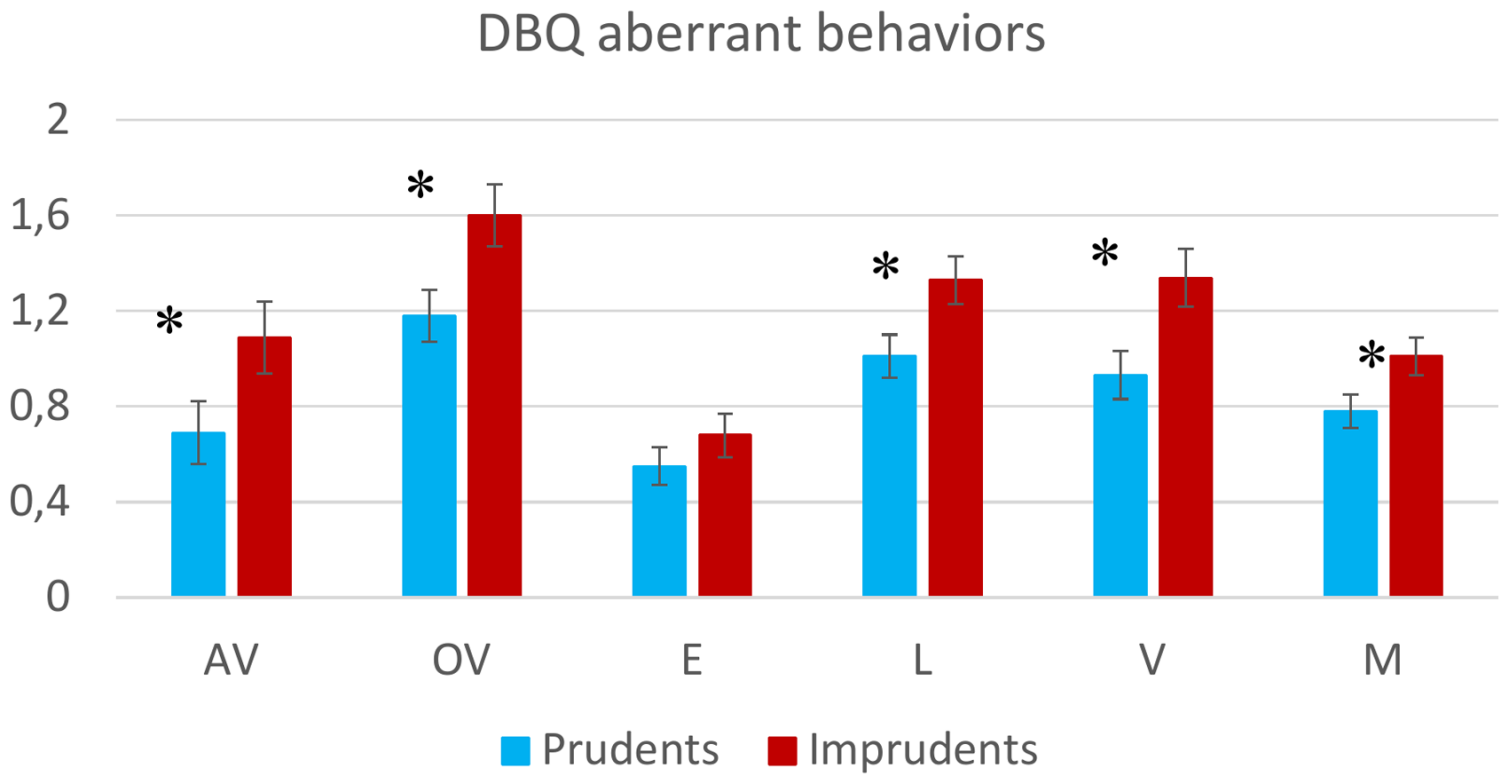
The figure shows the trends of Prudent and Imprudent participants with reference to the DBQ scores. Vertical bars represent standard errors. Asterisks indicate significant differences.

In addition, the results at the univariate level also show significant differences between the two clusters in terms of all the dependent variables concerning DBQ scores, with the exception of the E score (see Figure 5).

The results seem to reflect how Violations and Mistakes (at least in terms of Lapses) are related to risky driving behavior. This statement is consistent with results from over-speeding variables and with the rationale underlying the DBQ’s structure and theoretical basis. In fact, the DBQ questionnaire variables are derived from the individuation of items that refer to driving actions linked to acceleration, speeding, and over-speeding. The highlighted coherence between the two instruments seems to indicate that the self-report questionnaire is robust, despite the well-known limitations of these kinds of instruments, especially related to social desirability.

Particularly, the items that mainly support this consideration are item 10, “Non rispettare i limiti di velocità su una strada residenziale” [Disregard the speed limit on a residential road] (Smorti and Guarnieri, 2016, p. 17), and item 20, “Partire a tutta velocità davanti a un semaforo con l’intenzione di ‘battere’ il conducente accanto” [Race away from traffic lights with the intention of beating the driver next to you] (Smorti and Guarnieri, 2016, p. 18), included in the Ordinary Violations sub-scale, as well as item 16, “Arrabbiarti con un altro guidatore e inseguirlo per dirgliene quattro” [Become angered by another driver and give chase with the intention of giving him/her a piece of your mind] (Smorti and Guarnieri, 2016, p. 17), included in the Aggressive Violations sub-scale, which relates to over-speeding. On the other hand, regarding overall Mistakes, item 14, “Cercare di ripartire in terza da un semaforo” [Attempt to drive away from the traffic lights in third gear] (Smorti and Guarnieri, 2016, p. 17), and item 1, “Scontrarsi con un ostacolo che non avevi visto durante una svolta” [Hit something when reversing that you had not previously seen] (Smorti and Guarnieri, 2016, p. 17), included in the Lapses sub-scale, may refer to speeding and over-speeding, even though these items detect aspects related to failure in cognitive skills that would prevent previously planned actions from being carried out correctly. The fact that in the study by Parker et al. (1995) no relations between lapses and accidents were found may indicate that, if the speeding tendency detected through the simulator is not due to failure in action planning, it does not necessarily lead to a crash.

The absence of significant effects in error scores is in line with previous considerations concerning the DBQ’s theoretical basis (Parker et al., 1995) and the correlation results set out above. In fact, the items included in the Errors sub-scale do not refer strongly to imprudent behavior in terms of speed, acceleration, and over-speeding. Consequently, these items do not constitute basic information for understanding the phenomenon of risky driving due to celeration behaviors. On the contrary, the items included in this sub-scale mainly reflect failures in action planning due to misinterpretation of a problem or its solution.

## Conclusion

The results of this study show that information obtained from DBQ scores and HRT simulator variables are important and coherent in conveying information related to safe or unsafe driving habits, thus supporting the predictive value and suitability of the self-report questionnaire instrument. Despite the fact that the generalizability of the results from simulated driving studies has not yet been sufficiently demonstrated, the convergence of evidence of correspondence between recorded road violations and DBQ violation scores (de Winter et al., 2015) and between simulated driving and DBQ responses supports the usefulness of the questionnaire for driving style assessment.

The highlighted correspondences between the actual and objective driving performance variables and the participants’ subjective perceptions of their behavior on the road provide further evidence about the robustness of the self-report questionnaire. Consistent with the research hypotheses, comparing self-report questionnaire responses and driving-simulator performance supports previous insights about the correspondences between individual attitudes and driving behavior. Thus, these results are relevant to the integration of useful information in expanding interventions and training designed to reduce risky behavior and promote road safety. Indeed, as highlighted in the systematic review by Faus et al. (2021), studies revealed that intervention programs are more effective when several different countermeasures, such as educational programs, simulator-based driver trainings, improvement in legislation, and increase in police control, are associated. Moreover, communication strategies employed in advertising campaigns have different outcomes on different samples of population, being less effective in drivers more prone to risky behaviors, that is, those road users whose behavioral change would be most desirable (Faus et al., 2021). Furthermore, the recall of these kinds of intervention seems very low in some countries, and varies on the basis of educational level, age, sex, and income (Alonso et al., 2021), which suggest the need to replace the intervention on the basis of follow-up studies. For this reason, the availability of an easy-to-administer questionnaire for identifying the subsamples of population that behave riskier while driving is crucial to direct correctly tailored interventions and to monitor their effectiveness over time.

This article emphasizes the potential usefulness of virtual driving and self-report questionnaires for planning interventions targeted to specific kinds of road users according to their driving performances and related self-evaluation of their own driving abilities. In other words, to reduce traffic violations, characterized by over-speeding and abrupt changes in acceleration, it is appropriate to focus on changing drivers’ beliefs, norms, and attitudes toward road laws concerning the speed limits. This kind of intervention will be more effective when oriented to drivers with driving profiles characterized by over-speeding and high scores in the violation sub-scale of the DBQ.

Alternatively, attention must be devoted to driving skills to reduce Mistakes, as these aberrant driving behaviors imply cognitive failures in information processing. Thus, the possibility offered by simulators and questionnaires to identify drivers who suffer from this kind of impairment may be beneficial in helping to involve these drivers in training interventions aimed to improve action control and planning abilities concerning specific vehicle commands, such as the proper use of brakes, and to enhance conscientiousness in decision-making to avoid distraction while driving.

In conclusion, the present results point out the appropriateness and relevance of the DBQ self-report questionnaire, with particular emphasis on Ordinary Violations and Lapses, to analyze and develop further conceptualization with regard to driving behavior in terms of over-speeding trends. The limitations of this instrument depend on its structure as a self-report questionnaire, which, considering participants’ subjective perceptions, can lead to different information in comparison with that conveyed by objective performance on the road. These differences may be due to modulating variables such as self-esteem, suggestibility, respondents’ ability to interpret the items correctly, existing stereotypes, and individuals’ social desirability bias. However, the fact that the information obtained from the questionnaire is in line with that obtained by observing the simulated driving performance of participants is reassuring with respect to its possible usefulness, both for increasing the understanding of driving behavior and to plan proper interventions tailored to drivers’ characteristics. As highlighted in the introduction, the limitations in simulator validity and predictive ability need to be taken into account. Also, the fact that the sample of the present study is focused on a narrow range of ages and that participants are voluntary represent possible limitations, which need to be addressed in further studies. Nevertheless, as pointed out by Parker et al. (1995), all the methodologies employed in studies on driver behavior suffer of some limitations, such as ethical issues related to safety in on-road studies, social desirability in research based on self-report data, statistical constraints in studies using crash databases due to the fact that accidents are no so frequent (de Winter et al., 2015). Anyway, despite the necessary caution as to the generalizability of the present results to the road context, the coherence of the present findings concerning the relation between self-report measures and studies focusing on accidents and road infractions (Parker et al., 1995; de Winter et al., 2015) is comforting.

This paper may provide useful insights for the conceptualization and design of training and learning practices in the field of road safety that take driving behavior’s holistic nature into account. In this way, greater emphasis can be placed on the reciprocal complementarity of subjective and objective factors, internal and external to the person, to provide targeted interventions to reduce accidents and promote road safety.

## Data availability statement

The raw data supporting the conclusions of this article will be made available by the authors, without undue reservation.

## Ethics statement

The studies involving human participants were reviewed and approved by the Ethical Committee for the Psychological Research of the University of Padua. The patients/participants provided their written informed consent to participate in this study.

## Author contributions

MT: study conception and design. AG: data collection. MT and AG: analysis and interpretation of results, draft manuscript preparation, correction, and revision. All authors contributed to the article and approved the submitted version.

## Conflict of interest

The authors declare that the research was conducted in the absence of any commercial or financial relationships that could be construed as a potential conflict of interest.

## Publisher’s note

All claims expressed in this article are solely those of the authors and do not necessarily represent those of their affiliated organizations, or those of the publisher, the editors and the reviewers. Any product that may be evaluated in this article, or claim that may be made by its manufacturer, is not guaranteed or endorsed by the publisher.
